# Adsorption of cadmium and lead onto live and dead cell mass of *Pseudomonas aeruginosa*: A dataset

**DOI:** 10.1016/j.dib.2018.04.014

**Published:** 2018-04-10

**Authors:** Mahnaz Karimpour, Seyed Davoud Ashrafi, Kamran Taghavi, Ali Mojtahedi, Esmaeil Roohbakhsh, Dariush Naghipour

**Affiliations:** a School of Health, Guilan University of Medical Sciences, Rasht, Iran; b Research Center of Health and Environment, Guilan University of Medical Sciences, Rasht, Iran; c Department of Microbiology, Faculty of Medicine, Guilan University of Medical Sciences, Rasht, Iran

**Keywords:** Adsorption, Lead, Cadmium, *Pseudomonas aeruginosa*

## Abstract

In this research heavy metals, Cd and Pb, adsorption efficiency was evaluated in aqueous solutions using live and dead biomass of *Pseudomonas aeruginosa* bacteria. The various important parameters including; pH, temperature, Cd and Pb concentrations, contact time, live and dead cell mass were examined. First, the resistant *P. aeruginosa* to heavy metals identified and isolated from contaminated soil. Then, the Minimum Inhibitory Concentration (MIC) of Cd and Pb was determined for *P. aeruginosa*. The highest adsorption efficiency for Cd and Pb were 87% and 98.5%, under dead cell mass of 125 mg, pH 7, temperature 35 °C and contact time 90 min, respectively. The results of this study showed that *P. aeruginosa* have a high ability to adsorption of Cd and Pb in aqueous solutions.

**Specifications Table**

**Table:** 

**Subject area**	Environmental Engineering
**More specific subject area**	Adsorption process
**Type of data**	Figure and table
**How data was acquired**	Cd and Pb were measured by Atomic adsorption (Varian AA 220).
	PH was measured using the digital pH meter (Metrohm).
	PCR For final confirmation *Pseudomonas aeruginos* was done after biochemical tests.
	Temperature was measured by digital thermometer, (Testo).
**Data format**	Raw, analyzed.
**Experimental factors**	The effects of various factors including pH, contact time, temperature, heavy metals concentrations and live and dead cell mass concentrations of *Pseudomonas aeruginosa* on adsorption efficiency were investigated in batch system.
**Experimental features**	Different concentrations of live and dead cell mass of *Pseudomonas aeruginosa* and Cd and Pb, different pH, temperature and time, was used and adsorption efficiency was calculated.
**Data source location**	Department of Environmental Health Engineering, school of Health, Guilan University of Medical Sciences, Rasht, Iran.
**Data accessibility**	The data are available within this paper.

**Value of the data**

• The data suggest biological adsorption method to removal of heavy metals from soil and aqueous solution.
• This data can be used for development of adsorption system for removal of heavy metals from soil, water and wastewater.
• This data will be useful for the engineers which is associated with biological purification of water, wastewater and soil refinement.

## 1 Data

**Fig. 1:**
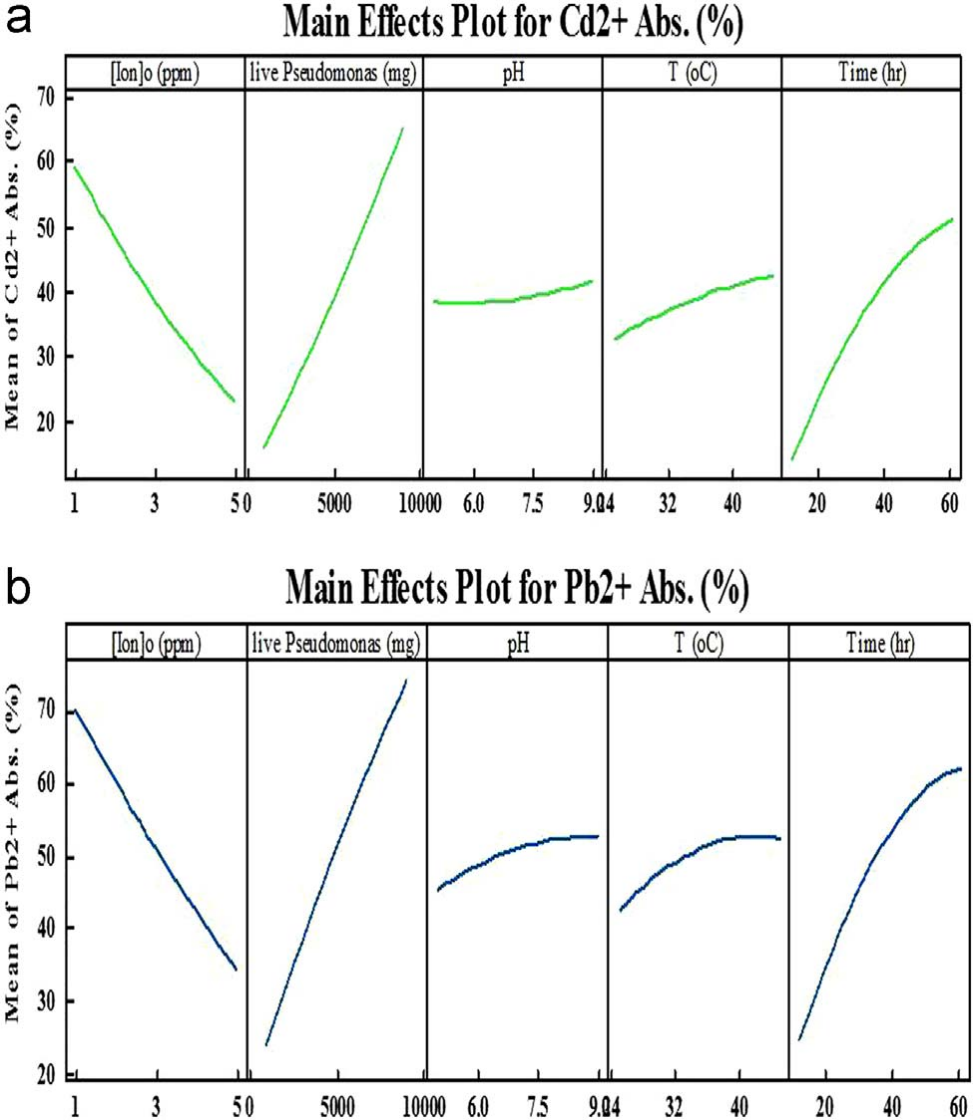
The main effects curve for cadmium (a) and lead (b) adsorption by dead cell mass of *Pseudomonas aerogenosa*.

**Fig. 2:**
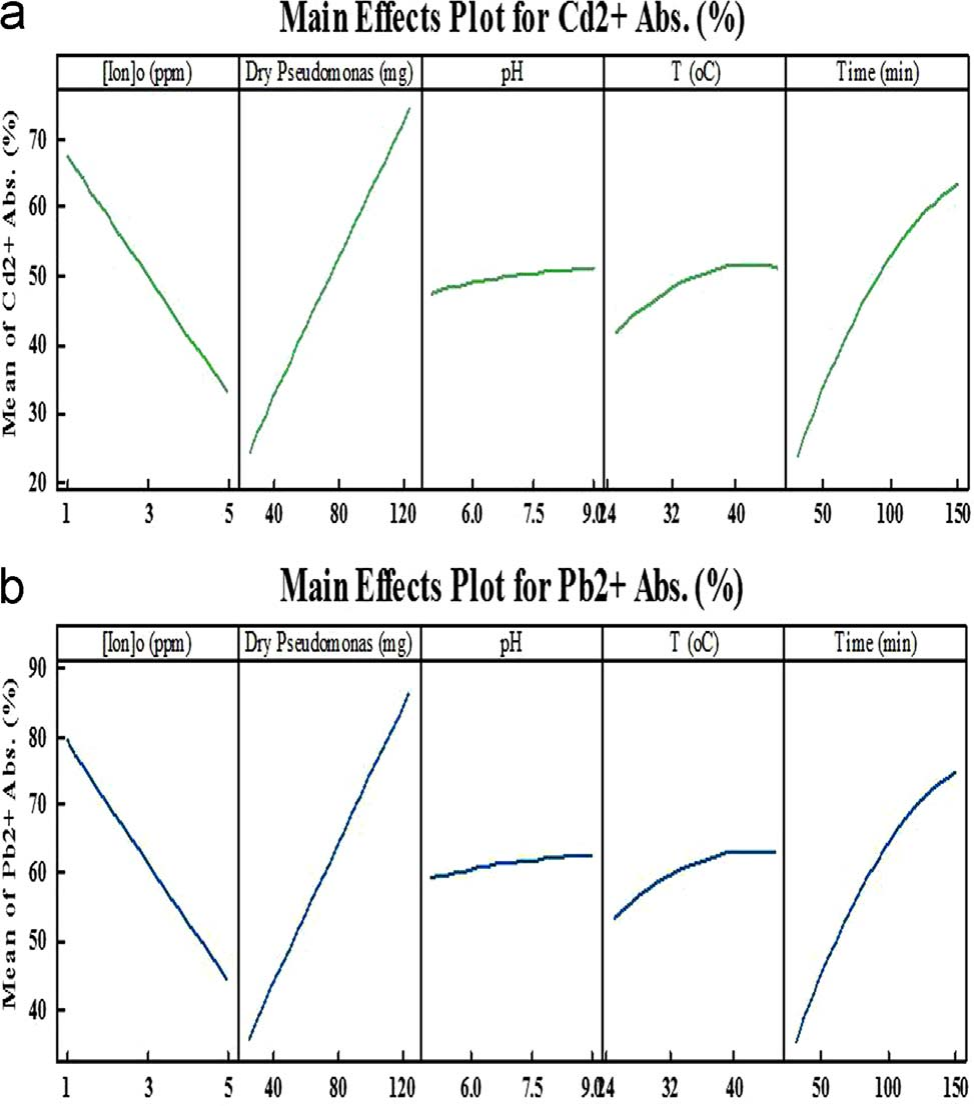
The main effects curve for the percentage of cadmium (a) and lead (b) adsorption by live cell mass of *pseudomonas aerogenosa*.

Environmental pollutants including heavy metals and organic maters has become an issue of severe international concern in recent years [1], [2], [3], [4], [5], [6], [7], [8]. The various process can be used for removal of these pollutants from the environment like adsorption systems [9], [10], [11], [12], [13], [14]. The agricultural soil and groundwater of Guilan Province, north Iran, contains high levels of Cd and Pb. In this work *Pseudomonas aeruginosa* bacteria isolated from agriculture soil. The various important parameters including; pH, temperature, Cd and Pb concentrations, contact time, live and dead cell mass were examined [14], [15], [16], [17]. The range of Pb and Cd adsorption percentages by using live and dead cell mass of *P. aeruginosa* are provided in Table 1, Table 2, Table 3, Table 4, Table 5 at different conditions. The highest adsorption efficiency for Cd and Pb were 87% and 98.5%, under dead cell mass of 125 mg, pH 7, temperature 35 °C and contact time 90 min, respectively. The main effects curve for lead and cadmium adsorption by live and dead cell mass of *Pseudomonas aerogenosa* were provided by Minitab 15 software and were showed in Fig. 1, Fig. 2.

**Table 1:** The range of Pb and Cd adsorption percentages at different pH by using dead cell mass of *Pseudomonas aeruginosa* (dead cell mass 75 mg, temperature 35 °C, contact time 90 min).

**pH**	**Cd (ppm)**					**Pb (ppm)**				
	**1**	**2**	**3**	**4**	**5**	**1**	**2**	**3**	**4**	**5**
**5**	63.9	55.7	47.6	39.7	31.9	75.3	67.1	59.2	51.6	44.1
**6**	66.0	57.4	49.0	40.7	32.6	77.3	68.8	60.5	52.4	44.5
**7**	67.7	58.8	50.1	41.5	33.1	79.1	70.2	61.5	53.0	44.7
**8**	69.0	59.8	50.8	41.9	33.1	80.7	71.3	62.2	53.3	44.6
**9**	70.0	60.5	51.1	41.9	33.0	81.9	72.2	62.6	53.3	44.2

**Table 2:** The range of Pb and Cd adsorption percentages at different temperatures by using dead cell mass of *Pseudomonas aeruginosa* (dead cell mass 75 mg, pH7, contact time 90 min).

**T**^**(OC)**^	**Cd(PPM)**					**Pb(PPM)**				
	**1**	**2**	**3**	**4**	**5**	**1**	**2**	**3**	**4**	**5**
**25**	64.9	53.4	42.0	30.8	19.7	76.6	65	53.6	42.4	31.4
**30**	67.1	56.9	46.9	37.0	27.2	78.7	68.4	58.3	48.5	38.9
**35**	67.7	58.8	50.1	41.5	33.1	79.1	70.2	61.5	53.0	44.7
**40**	66.5	59.0	51.6	44.3	37.2	78.0	70.4	63.0	55.9	48.9
**45**	63.6	52.4	51.3	45.4	39.6	75.2	68.9	62.9	57.1	51.5

**Table 3:** The range of Pb and Cd adsorption percentages at different times by using dead cell mass of *Pseudomonas aeruginosa* (dead cell mass 75 mg, temperature 35 °C, pH7).

**Time**_**(min)**_	**Cd(PPM)**					**Pb(PPM)**				
	**1**	**2**	**3**	**4**	**5**	**1**	**2**	**3**	**4**	**5**
25	40.8	30.9	21.2	13.6	5.1	52.5	42.7	32.9	23.2	14.0
50	52.9	43.4	34.1	24.9	15.9	64.5	54.9	45.5	36.4	27.5
75	62.8	53.7	44.7	35.9	27.3	74.3	65.1	56.2	47.4	38.9
100	70.5	61.7	53.2	44.7	36.5	81.9	73.1	64.6	56.2	48.1
125	75.9	67.5	59.4	51.3	43.4	87.4	79.0	70.8	62.8	55.1
150	79.1	71.1	63.3	55.6	48.1	90.6	82.6	74.8	67.3	59.9

**Table 4:** The range of Pb and Cd adsorption percentages at different dead cell mass by using dead cell mass of *Pseudomonas aeruginosa* (temperature 35 °C, pH7, contact time 90 min).

**Dead cell mass(mg)**	**Cd(PPM)**					**Pb(PPM)**				
	**1**	**2**	**3**	**4**	**5**	**1**	**2**	**3**	**4**	**5**
**30**	49.0	38.0	27.1	16.3	5.8	60.7	49.5	38.5	27.7	17.1
**50**	57.4	47.3	37.5	27.7	18.0	69.0	58.8	48.8	39.0	29.5
**70**	65.6	56.5	47.6	45.2	30.1	77.1	67.9	59.0	39.8	41.7
**90**	73.6	66.9	57.5	49.6	41.9	85.1	75.5	68.9	61.2	53.7
**110**	81.4	74.2	67.2	60.0	55.5	92.9	85.7	78.7	72.0	53.5
**125**	87.0	80.6	74.3	68.1	62.1	98.5	92.1	86.0	80.0	74.3

**Table 5:** The range of Pb and Cd adsorption percentages at different live cell mass by using living cell (temperature 35 °C, pH7, contact time 36 h).

**Live cell mass(gr)**	**Cd(PPM)**					**Pb(PPM)**				
	**1**	**2**	**3**	**4**	**5**	**1**	**2**	**3**	**4**	**5**
**2**	44.3	32.2	21.3	11.7	10.5	53.3	41.8	30.9	20.8	11.3
**4**	53.9	42.7	32.8	24.0	16.5	64.9	54.4	44.6	33.4	27.0
**6**	64.4	54.1	45.0	37.2	30.6	75.5	66.0	57.2	49.1	41.6
**8**	75.7	66.3	58.1	51.2	45.5	85.0	76.5	68.7	61.6	55.2
**9**	81.6	72.7	62.0	58.5	53.3	89.4	81.4	74.1	67.5	61.6

## 2 Materials and methods

### 2.1 Materials

All biological and chemical materials used in the experimental, were provided with degree of purity and purchases has been done Merck Company, Germany.

### 2.2 Isolation of *Pseudomonas aeruginosa* and experiments

First, the agricultural soil samples from Guilan province were prepared at different dilutions and cultured in the cultivation environments nutrient agar and Mac Conkey agar and for 24 h incubated in the temperature 37 °C and several steps repeated until prepared a pure culture. Further biochemical tests done for recognize that include:

**Catalase Test:** Use a loop or sterile wooden stick to transfer a small amount of colony growth in the surface of a clean, dry glass slide. Place a drop of 3% H2O2 in the glass slide observe for the evolution of oxygen bubbles.

**Lactose test:** some of colony was inoculated to tubes contain lactose broth.

**Sim test:** H2S, indole, Motility was at *P. aeruginosa*.

**Urease test:** Rapid urease-positive organisms turn the entire medium pink within 24 h.

**TSI test:** 0.1% Glucose, 1.0% lactose/1.0% sucrose: Iron, was at *P. aeruginosa*.

**Gram staining:** Negative gram.

This bacteria in the cultivation environment MHA produced blue pigment.

**Fig. 3:**
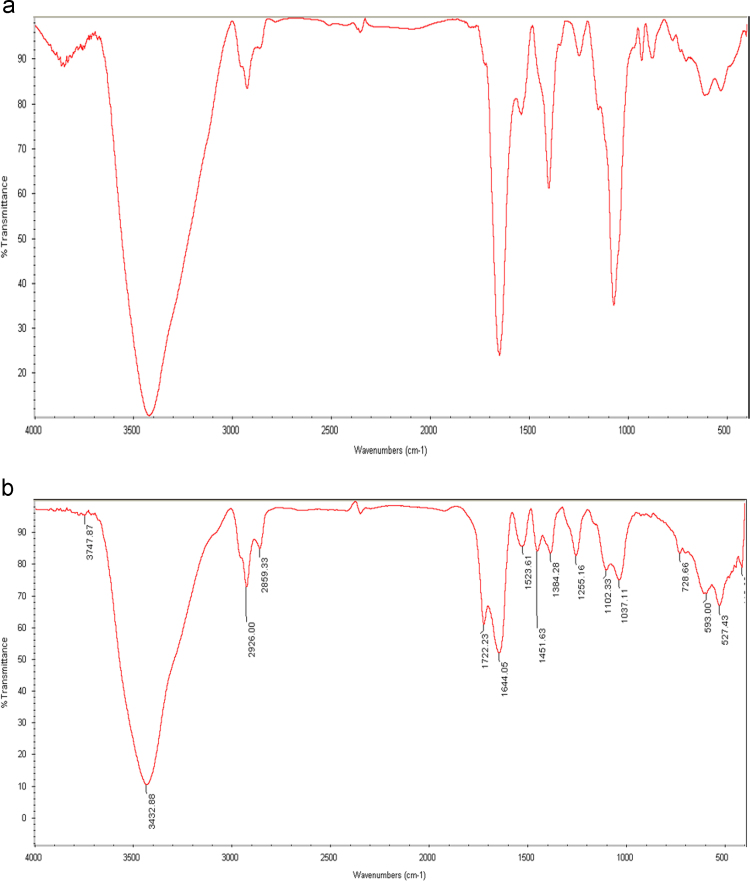
The FTIR of live (a) and dead (b) cell mass of *pseudomonas aerogenosa*.

For final approval isolated bacteria used from PCR method with characteristics down:

Characteristics of primers were presented for *P. aeruginosa* in Table 6. Also the FTIR of the live and dead cell mass was prepared and presented in Fig. 3.

**Table 6:** Primers sequences used to detect 16SrRNA *Pseudomonas aeruginosa.*

**PCR method**	**Primer(5′ to 3′)**	**Anneal temperature (°C)**	**DNA target**	**Accession in gen Bank**
Forward	GGGGGATCTTCGGACCTCA	58	16SrRNA	AB091760.1
Reverse	TCCTTAGAGTGCCCACCCG			

For live cell mass preparing, after separating the bacteria from the soil, microorganism cultured in Erlenmeyer contain 100 ml lactose broth, incubated for 24 h at temperature 35 °C, 200 rpm. Then, it was centrifuged at 8000 rpm for 20 min and upper liquid was removed and the separated microorganism was used for adsorption process. For dead cell mass preparing, microorganism cultured in Erlenmeyer contain 100 ml lactose broth, incubated for 24 h at temperature 35 °C, 200 rpm. Then it was centrifuged at 8000 rpm for 20 min and upper liquid was removed and the residual of it dried at 60 °C. To study of the adsorption process, live and dead cell mass was used in the batch system (250 ml Erlenmeyer), in different conditions (pH 5, 6, 7, 8, 9 and temperature 25, 30, 35, 40, 45 °C and Cd and Pb concentrations 1, 2, 3, 4, 5 mg/L and contact time 25, 50, 75, 100, 125, 150 min for dead cell mass and 10, 20, 30, 40, 50, 60 h for live dead mass). The data of heavy metals concentration in adsorption process were collected and the efficiency percent of adsorption was calculated by following equation;

$$\text{R}\;(\%)=100\;\times \;\frac{C_{0}-C_{t}}{C_{0}}$$

where *c*_0_ and *c_t_* are the concentrations of the primary and secondary heavy metals, respectively. Data analysis was performed using Minitab 15 software.

## Figures and Tables

**Figure:**
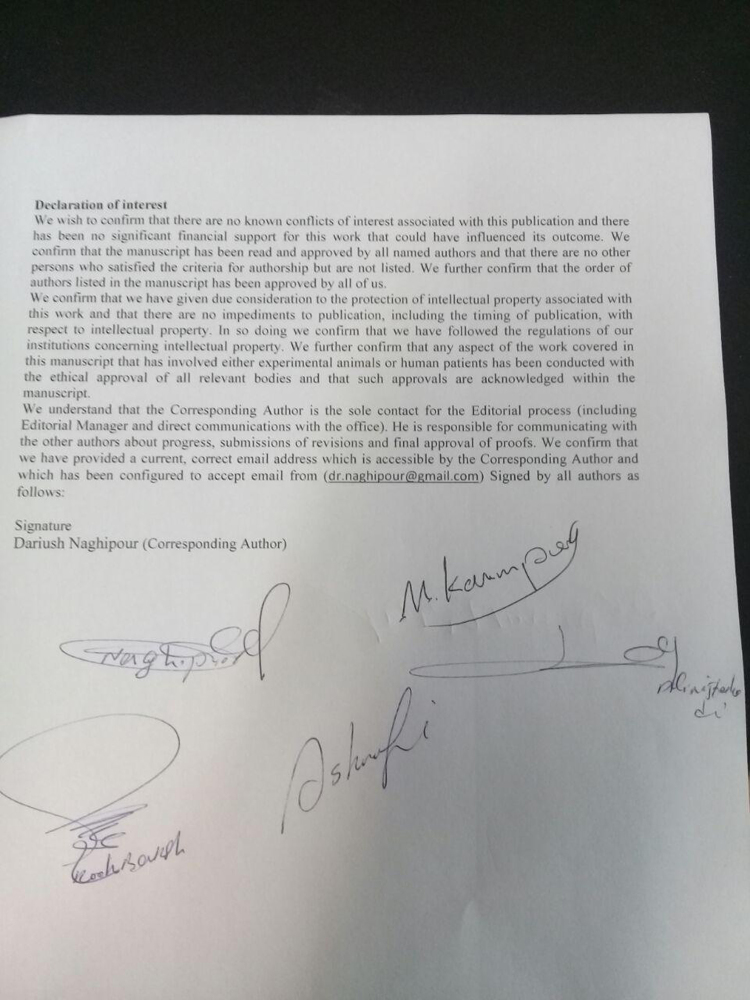
Supplementary Fig. S1.
